# Compartmentalization of membrane trafficking, glucose transport, glycolysis, actin, tubulin and the proteasome in the cytoplasmic droplet/Hermes body of epididymal sperm

**DOI:** 10.1098/rsob.150080

**Published:** 2015-08-26

**Authors:** Catherine E. Au, Louis Hermo, Elliot Byrne, Jeffrey Smirle, Ali Fazel, Robert E. Kearney, Charles E. Smith, Hojatollah Vali, Julia Fernandez-Rodriguez, Paul H. G. Simon, Craig Mandato, Tommy Nilsson, John J. M. Bergeron

**Affiliations:** 1 Department of Anatomy and Cell Biology, McGill University, Montreal, Quebec, CanadaH3A 0C7; 2 Department of Medicine, McGill University Health Centre Research Institute, 1001 Decarie Blvd, Montreal, Quebec, CanadaH4A 3J1; 3 Department of Biomedical Engineering, McGill University, Montreal, Quebec, CanadaH3A 1A1; 4 Centre for Cellular Imaging, Sahlgrenska Academy at the University of Gothenburg, PO Box 435, 40530 Gothenburg, Sweden; 5 Royal Victoria Hospital, Center for Translational Biology, RI-MUHC, Glen Site, 1001 Decarie Blvd, Bloc E, Room E02.7210, Montreal, Quebec, CanadaH4A 3J1

**Keywords:** membrane traffic, cryosection electron microscopy localization, subcellular fractionation, quantitative proteomics, light microscope localization

## Abstract

Discovered in 1909 by Retzius and described mainly by morphology, the cytoplasmic droplet of sperm (renamed here the Hermes body) is conserved among all mammalian species but largely undefined at the molecular level. Tandem mass spectrometry of the isolated Hermes body from rat epididymal sperm characterized 1511 proteins, 43 of which were localized to the structure *in situ* by light microscopy and two by quantitative electron microscopy localization. Glucose transporter 3 (GLUT-3) glycolytic enzymes, selected membrane traffic and cytoskeletal proteins were highly abundant and concentrated in the Hermes body. By electron microscope gold antibody labelling, the Golgi trafficking protein TMED7/p27 localized to unstacked flattened cisternae of the Hermes body, as did GLUT-3, the most abundant protein. Its biogenesis was deduced through the mapping of protein expression for all 43 proteins during male germ cell differentiation in the testis. It is at the terminal step 19 of spermiogenesis that the 43 characteristic proteins accumulated in the nascent Hermes body.

## 1. Introduction

Discovered in 1909 [1], the cytoplasmic droplet of epididymal sperm is seen as a small bulge of cytoplasm (2 µm in diameter) positioned at the neck or connecting piece of the flagellum [2–4]. The presence and migration of the cytoplasmic droplet along the sperm flagellum from the neck to the annulus [2,5,6] occur in proximal regions of the epididymal duct where motility is initiated [7,8].

By electron microscopy (EM) of the droplet, flattened unstacked cisternae are abundant, which, along with dilated and elongated membranous profiles and vesicles of different sizes, concentrate and segregate to one pole of the spherical structure [9]. The flattened cisternae of the cytoplasmic droplet are derived from the fragmentation and unstacking of the Golgi apparatus of late spermatids, as first deduced by morphology and Golgi protein localization [9], and later confirmed by Moreno *et al.* [10].

The cytoplasmic droplet is needed to initiate motility as deduced from a comparison of sperm with or without droplets [5]. By mass spectrometry of five bands excised from SDS PAGE of isolated cytoplasmic droplets, 105 largely soluble proteins (including glycolytic enzymes) were characterized that were proposed to regulate the initiation of motility through the generation of ATP [11]. However, the major morphological structures in the droplet, including the characteristic flattened internal membranes [9], were not retained in the isolated structure and integral membrane proteins were largely missing.

The biogenesis of the cytoplasmic droplet occurs in the testis at the last step of germ cell differentiation, step 19 of spermiogenesis [9]. Testis-expressed proteins affect droplet formation. For example, spermatid maturation 1 (Spem1) expressed in late steps of germ cell differentiation in the testis is needed for normal droplet formation and fertility [12]. The *15Lox* gene that expresses organelle degradation enzyme, 15-lipoxygenase in male germ cells of the testis, is required for epididymal sperm maturation, droplet migration and morphology, normal fertility, and normal litter size [13]. With respect to the Golgi origin of the internal flattened cisternae of the structure, a recent detailed analysis of Golgi apparatus in differentiating germ cells of the testis revealed a subset of Golgi proteins in the forming structure at step 19 [14]. However, the contribution of proteins from other membrane sources and other cellular structures was not assessed. This was the objective of this study.

Using a procedure that retains the internal membranes of the isolated cytoplasmic droplet and a methodology for quantitative protein characterization of all proteins including integral membrane proteins by tandem mass spectrometry, 1511 abundant proteins have been characterized in this study. Through antibody-based localizations of 58 proteins, their expression during germ cell differentiation in the testis could be compared with localization to the cytoplasmic droplet of sperm in the epididymis, including the 1318 proteins characterized previously for male germ cell Golgi apparatus [14].

Based on these data, the hypothesis that the cytoplasmic droplet originates in the late spermatid of the testis to coordinate membrane trafficking with the initiation of sperm motility in the epididymis is supported. We propose that the cytoplasmic droplet be renamed the Hermes body. Hermes is a winged god of transitions and boundaries, and with a physical attribute to male virility. The Hermes body is deduced to regulate, through its makeup of enzymes and internal membranes and cytoskeletal constituents, the transition of an immotile, unfertile to motile, fertile sperm in the epididymis.

## 2. Material and methods

### 2.1. Animals

All animals used in this study were maintained on a 12 L/12 D cycle in the animal facility and fed *ad libitum*. The procedures for animal use were done in accordance with the guidelines of the McGill Animal Care Committee.

### 2.2. Hermes body isolation and characterization

Adult Sprague Dawley rats (350–450 g) were purchased from Charles River Laboratories Canada (St Constant, Quebec, Canada). For each isolation procedure, 10 rats were used. After anaesthesia, the testes were removed from the scrotum following intracardiac saline perfusion. The epididymis was removed from the testis by cutting through the rete testis. The initial segment, caput and corpus down to its mid area were removed and placed in ice-cold buffer (50 mM Tris–HCl pH 7.4, 25 mM KCl, containing either 1 mM PMSF, 200 K units of aprotinin per millilitre of buffer and 1 µM leupeptin per millilitre of buffer or 0.5 mM Pefabloc; Sigma). The epididymides were removed from the buffer, placed on a paraffin block on ice and diced into 1 mm pieces. Diced epididymides were placed in a Falcon tube with 15–20 ml of ice-cold buffer and then vortexed at medium to high speed 5 × 10 s, being returned to the ice for a few seconds between each vortex. The suspension was poured through a metal sieve and then through a 150 µm Nitex net to remove connective tissue. A milky suspension of sperm was collected in a 30 ml Corex tube and centrifuged at 1500*g* for 15 min (Avanti rotor R-20, 3500 r.p.m. (1500*g*). The pellet (P1) was put aside on ice. The supernatant (S1) was centrifuged at 150 000*g* for 15 min at 4°C (Ti60 rotor, 36 000 r.p.m., 100 000*g*). The supernatant (S2) was discarded. The pellet (P2) was combined with P1, and re-suspended in 5 ml of ice-cold buffer with a glass rod. The suspension was passed through a 20 gauge needle five times, and then centrifuged at 150*g* for 10 min at 4°C to give a pellet (P3) and supernatant (S3). S3 (4.5 ml) was placed above a sucrose step gradient made of 2 ml each of 0.6 M, 0.8 M, 1.0 M and 1.2 M sucrose (in ice-cold buffer), with the refractometer readings 19.2, 25.0, 31.0 and 35.5, respectively. This was centrifuged for 90 min at 40 000 r.p.m. in an SW-40 rotor (202 000*g*) with the brake on. The interface between 0.8 and 1 M sucrose (fraction 3) was collected with a needle and syringe and designated the cytoplasmic droplet/Hermes body fraction.

Fraction 3 was enriched in the Golgi marker enzyme UDP-galactose ovomucoid galactosyl transferase 61.3-fold ± 22.5 (mean ± s.d., *n* = 4) over the sperm homogenate.

### 2.3. Routine electron microscopy processing of *in situ* epididymis and isolated subcellular fractions

For routine EM analysis, four adult male Sprague Dawley rats were anaesthetized with sodium pentobarbital and their epididymides fixed by perfusion through the abdominal aorta with 2.5% glutaraldehyde in 0.2 M sodium cacodylate buffer containing 0.1% calcium chloride, pH 7.4 [2]. After fixation (10 min), the tissue was removed, trimmed and left in fixative for 2 h. It was then postfixed in potassium ferrocyanide reduced osmium tetroxide for 1 h, after which it was dehydrated in alcohol, acetone and embedded in Epon. Sections were cut with a diamond knife and stained with uranyl actetate and lead citrate, and examined with a Philips 400 EM, Tecnai 12 120 kV TEM and Tecnai G2 F20 Cryo-STEM (FEI Inc.).

For isolated Hermes bodies, portions of samples from *n* = 3 isolated fractions were processed for EM analysis. Two millilitre of isolated subcellular fractions (100–150 µg protein) in ice-cold buffer were mixed with 2 ml of fixative (5% glutaraldehyde in 0.2 M sodium cacodylate buffer containing 0.1% calcium chloride, pH 7.4) on ice in a chemical fume hood. The Hermes body fractions were collected onto filter membranes (Millipore nitrocellulose filter 0.45 mm HA), washed with 0.1 M cacodylate buffer (pH 7.4 containing 5% sucrose) three to four times, incubated with tannic acid (1% tannic acid Mallinckrodt CN No 1764 in 0.1 M cacodylate buffer pH 7.4) for 1 h, washed with cacodylate buffer containing 1% sodium sulfate, placed in 100 mM maleate buffer pH 5.7, incubated with uranyl acetate (6% uranyl acetate in 0.1 M sodium maleate, pH 5.7) for 2 h on ice in the fume hood, washed with maleate buffer, dehydrated through a series of graded alcohols (70%, 80%, 90%, 95% and 100% ethanol) for 10 min for each step, and then dissolved in 100% acetone for 1–2 h. The samples were embedded in Epon resin and the blocks were then cut and sectioned (90–100 nm), stained with uranyl acetate and lead citrate and examined with a Tecnai 12 120 kV TEM equipped with a Gatan 792 Bioscan CCD Camera (Gatan Inc., Pleasanton, CA, USA).

### 2.4. Electron microscope tomography

Samples were prepared as described above. However, thicker sections (approx. 250 nm) were cut for electron tomography and transferred onto carbon-coated copper grids. Images were collected on a Titan Krios microscope operated at 300 kV using a Gatan Ultrascan 4kx4 k CCD camera. For electron tomography, data collection was done at an electron dose of approximately 1500 electrons/Å2 per tomogram. Focusing was done on an adjacent area in order to minimize electron dose exposure. In total, over 30 tomograms were collected at different magnifications ranging between 20 and 50 k. Tilt series were taken using the FEI software in the angular range between −64° and +64° with 2o increments. For the estimated sample thickness, this would be sufficient for a resolution of 2 nm, following the Crowther formula. Reconstruction of three-dimensional volumes was done using the IMOD software suite [15]. The final tomograms were binned three times in order to increase the signal-to-noise ratio. Three-dimensional rendering was done using Chimera [16].

### 2.5. Tandem mass spectrometry

The methodologies described by Gilchrist *et al.* [17] and Bell *et al.* [18] were followed. The raw data were processed in pipeline format [19] to generate a peaklist of all tandem MS by employing Distiller followed by Mascot Cluster. The Mascot search results were then parsed into the in-house relational database termed CellMapBase [20], scored for protein identifications and grouped to present a minimum set of protein identifications [21] to account for all tandem MS assigned at 95% confidence. The concatenated peaklist was searched against a copy of the National Center for Biotechnology Information non-redundant database (ftp://ftp.ncbi.nih.gov/blast/db/FASTA/nr.gz) (release: NCBI nrdb 2008). For generation of the minimum list of proteins [21], protein identifications were grouped based on redundant peptide assignments taking into account redundancies that arise due to homologous sequences, truncated or partial sequences, alternatively spliced proteins, strain-specific allelic variation or redundant assignments of tandem MS. In this process, peptides were assigned as unique to an identification or shared between two or more identifications. For quantification, redundant peptide counting (spectral counts) was performed essentially as described previously [17,18,22,23]. In the present case, peptides were grouped to their cognate proteins based on the gel resolved sample and shared peptides were apportioned to the cognate proteins based on the proportion of unique peptides. For comparison among different subcellular fractions isolated, the percentage of total peptides for each of *n* = 3 or *n* = 4 biological repeats were averaged to represent the percentage total peptide counts for each subcellular fraction (electronic supplementary material, table S1).

### 2.6. Data analysis

As indicated above, all protein identifications made by peptides assigned by Mascot at the 95% confidence level (false positive rate approx. 1.5% estimated by searching a randomized copy of the database for the legacy data [17]) were tabulated into a relational database (CellMapBase) for further manipulations. The list of identified proteins is shown in the electronic supplementary material, table S1. The non-redundant list (electronic supplementary material, table S1) of proteins identified in this study was finalized as a consequence of curation (see below), first by literature search via the NCBI Gi number to categorize all identifications into 1 of 22 functional categories as described [17] and then to assign to each a representative protein name for equivalent proteins as determined by sequence identity [24]. Functional categories: biosynthetic cargo, blood + other cells, calcium transport/binding, coat, cytoskeleton, detoxification, GTPase, likely contaminants, lysosome, metabolism, mitochondria, nucleus, peroxisome, plasma membrane, proteasome/ubiquitin, protein modification, protein synthesis/folding, signalling, tethering/docking/fusion, traffic, trypsin and unknown.

### 2.7. Hierarchical clustering

Hierarchical clustering was done using the Cluster 3.0 program (http://bonsai.ims.u-tokyo ac.jp/∼mdehoon/software/cluster/) [25], and clusters were viewed by the Java Treeview program (http://jtreeview.sourceforge.net/) [26]. Excel was used for all data analysis.

### 2.8. Light microscope immunohistochemistry

The testes and epididymides of adult rats used for light microscope (LM)-immunohistochemistry (IHC) and in some cases LM-immunofluorescence (IF) were fixed by perfusion through the abdominal aorta for 10 min, after which the tissues were left overnight in fresh fixative [2] or by simple immersion in Bouin's for 24 h (*n* = 4 for each) at room temperature. Other rats were fixed by perfusion or immersion (*n* = 2 for each) with Zinc Fixative [27] (BD Biosciences, Cat 550523; Mississauga, Ontario, Canada).

Upon removal from the animal, the testes and epididymides were sectioned along their long axis to form two roughly equal halves. After fixation, all tissues were subsequently placed in 70% alcohol for several days before being dehydrated and embedded in paraffin.

Paraffin sections were cut at a 5 µm thickness and mounted on ‘Posi-Plus’ slides (Fisher Scientific Company; Ottawa, Ontario, Canada). Sections of both tissues were deparaffinized with Histoclear (Fisher brand 22-143975; Fisher Scientific) and rehydrated in a series of 100, 95, 80, 70 and 50% ethanol solutions, 0.3 M glycine, and phosphate-buffered saline (PBS).

Following rehydration, immunostaining was performed on Bouin-fixed tissue with the Envision+ System-HRP (DAB, diaminobenzidine) anti-rabbit Kit (Cat K4010; Dako Canada, Mississauga, Ontario, Canada) and a wash buffer solution containing 0.05 M Tris, 0.3 M NaCl and 0.1% Tween 20, pH 7.4. Dilutions for each primary antibody (using 5% bovine serum albumin (BSA) in PBS) were optimized and fell within a range of 1 : 100–1 : 500, and were incubated on the slides at room temperature for 1.5 h. Slides were washed 10 × 1 min and incubated with the secondary antibody (Envision+ kit), for 60 min at room temperature. The slides were again washed for 10 × 1 min, and incubated with the DAB solution from the Envision+ kit (time of incubation was optimized for each protein, and fell into the range of 5–30 s). The reaction product was visualized as a brownish colour with the intensity varying according to a given antibody. The sections were counterstained for 10 s in methylene blue, washed and quickly dehydrated through graded ethanol solutions to Histoclear. Coverslips were mounted onto the slides with Permount.

Negative controls for all experiments consisted of substituting PBS for primary antibody. Negative control experiments were performed using the above protocol, but without primary antibody. Non-immune sera (a preimmune antiserum to enthoprotin) were also substituted for primary antibody as control with no immunoreactivity detected. When available (e.g. anti-calnexin), preimmune sera were also tested and shown to have no detectable immunoreactivity.

### 2.9. Immunofluorescence

Sections were deparaffinized in hexane (Fisher Scientific), rehydrated in a graded ethanol series and washed in distilled water followed by 50 mM Tris-buffered saline (TBS), pH 7.4. Sections were incubated for 3 h at room temperature with primary antibody diluted as above in TBS. Sections were washed with TBST (TBS + 0.1% Tween 20), blocked for 20 min in a 2% casein solution and incubated for 30 min at room temperature with Alexafluor 594-labelled goat anti-rabbit IgG antibody (Invitrogen Canada) diluted 1 : 500 in TBST. Samples were washed with TBST, rinsed in TBS and counterstained for 1–3 min at room temperature with 300 nM 4′,6-diamidino-2-phenylindole dihydrochloride (DAPI; Invitrogen Canada) in TBS. Samples were rinsed in TBS, and coverslips were mounted using Prolong Gold antifade reagent (Invitrogen Canada). Sections were examined and photographed on a Zeiss Axioskop 2 motorized LM equipped with variable intensity FluorArc epifluoresence mercury lighting and AxioCam HR colour digital camera (Carl Zeiss Canada; Montreal, Quebec, Canada). Controls were done as indicated above.

For LM-IF of tissue sections, the results are based on 10 slides with an additional three as controls. Four antibodies were examined by LM-IF.

### 2.10. Electron microscopy immunogold labelling of epididymis

Two adult male Sprague Dawley rats were anaesthetized with sodium pentobarbital and their epididymides fixed by perfusion through the abdominal aorta with 0.5% glutaraldehyde and 4% paraformaldehyde in 0.1 M PBS (pH 7.4). After removal of the epididymis, the initial segment region was trimmed into small pieces (0.5 mm^3^), immersed for 2 h in the above fixative at 4°C, washed three times in 0.15 M PBS (pH 7.4), and then treated with PBS. Cryo-ultrathin sections of the small pieces of tissue were obtained following a modified procedure of Tokuyasu [28] and Zierold [29]. The tissue pieces were transferred onto a metal stub, rapidly frozen by plunging into liquid nitrogen, and stored in liquid nitrogen before cryo-ultramicrotomy. The stub was mounted on the specimen holder of an Ultracut-E ultramicrotome (Reichert) equipped with an FCS cryosystem. Cryo-ultrathin sections (70–100 nm thick) were cut from the frozen pieces in a cryo chamber at −70°C with a dry glass knife. The sections were picked up with a gold loop filled with a 2.3 M sucrose solution, transferred onto formvar-coated 200 mesh copper TEM grids, and kept on ice in a drop of PBS and 2.0% BSA/2% casein/0.5% ovalbumin (BCO).

Ultrathin sections of the initial segment of the epididymis were mounted on 300 mesh, formvar-coated nickel grids (Canemco, Montreal, Quebec, Canada). Each grid was floated for 5 min on a drop of 2% glycine in PBS, followed by 10 min on a drop of 2% bovine casein-ovo albumin (BCO), and then incubated for 1 h on 15 µl drops of primary rabbit antibodies to glucose transporter 3 (GLUT-3) and TMED7/p27 in 1 : 1, 1 : 5 and 1 : 10 dilutions with BCO. Sections were washed six times for 5 min each in PBS followed by a 2% BCO block for 5 min. The grids were then incubated for 20 min with a secondary goat anti-rabbit IgG antibody conjugated to 12 nm colloidal-gold (Jackson ImmunoResearch 1 : 20 dilution in BCO). The sections were subjected to six 5 min washes in PBS, followed by six 5 min washes in distilled water. Sections were counterstained with uranyl oxalic acetate stain (pH 7) in water for 5 min, followed by three 1 min washes with distilled water. Sections were protected with a 12 min incubation in a layer of 2% methylcellulose (Anachemia, Montreal, Quebec, Canada).

### 2.11. Quantification of gold decorated antibody labelling of cryosections of epididymal Hermes bodies

Electron microscopy of immunolabelled cryo sections was conducted using a Tecnai 12 120 kV TEM and Tecnai G2 F20 Cryo-STEM (FEI Inc.). Sections were randomly sampled with the only criteria being presence of gold particles in cross, longitudinal or oblique sections of the Hermes body. Micrographs were taken at magnification ranging from 30 000× to 60 000× with an AMT XR80C CCD Camera System on the Tecnai 12 and a Gatan Ultrascan 4000 4 k × 4 k CCD Camera System Model 895 on the Tecnai F20. Grids prepared with no primary antibody but incubated in gold-conjugated secondary antibody were used as controls.

A total of 10 sections were examined at different dilutions from each of two different animals and at five different dilutions. A total of 290 electron micrographs were analysed, of which 30 were controls.

Gold particle quantification was conducted as follows, with gold particles counted as individual particles when seen individually or when several were closely clustered together. To reveal gold particle distribution over the plasma membrane, longitudinal sections of the sperm flagellum were used. Gold particles in the area of the plasma membrane were scored over several demarcated 12 nm increments placed on either side of the plasma membrane (intra and extracellular). A 72 nm span (36 in each direction) on either side of the plasma membrane was determined as being the area where the most gold particles were positioned. This was determined for GLUT-3 from cross-sectional micrographs of the sperm flagellum where only plasma membrane surrounding an enclosed flagellum were found. To define a reference ‘membrane area’ 12 nm gold particles along a cross section of the flagellum were counted both intracellularly and extracellularly. Gold particles were counted individually, with each particle of clusters being counted as one. A 72 nm area with the flagellum's membrane at its centre was determined to be the area of maximum gold distribution along the cross section of the flagellum. This span of 72 nm was then used as a reference area for gold quantification in micrographs of Hermes bodies allowing classification of gold particles as being located in five distinct areas: cross and oblique sections of plasma membrane, intracytoplasmic (cisternae), and cross and oblique sections of flagellar components. The total number of gold particles was then counted in order to calculate percentage of gold distribution in each category on the various electron micrographs. Sections with no primary antibody were used as controls.

### 2.12. *In situ* sperm immunofluorescence

Epididymides were dissected from adult rats (*n* = 2), and sperm were removed from the caput and corpus regions. Immediately sperm were placed in fixative containing 2% paraformaldehyde and 0.2% glutaraldehyde in PBS for 45 min at room temperature. Samples were centrifuged at low speed (500*g* for 4 min at room temperature), and the supernatant was removed. Samples were washed (3 × 2 min in PBS), and the auto-fluorescence was quenched by incubating the samples in 50 mM ammonium chloride for 10 min at room temperature. The sperm were washed (3× 2 min in PBS), and then blocked and permeabilized for 45 min at room temperature in PBS containing 0.2% Fish Skin Gelatin (Sigma) and 0.05% Triton X-100. Samples were washed again, and incubated with antibodies to GLUT-3 (diluted 1 : 500 in PBS containing 0.2% fish skin gelatin) for 1 h at room temperature. After washing, the sperm were then incubated with goat anti-rabbit Alexa fluor 546 (diluted 1 : 1000) and phalloidin fluor 488 nm (Molecular Probes; diluted 1 : 200) for 45 min at room temperature. Samples were again washed, and finally incubated with DAPI for 2 min at room temperature. Following a final wash, the sperm were placed on a microscope slide, and coverslips were mounted using Prolong Anti-fade Gold mounting media (Invitrogen). Images were acquired using a LSM 700 series microscopy system (Carl Zeiss) fitted with a plan-apochromat 63×/1.40 oil immersion objective in sequential scanning mode with the pinhole set to obtain an optical section of about 0.8 µm in all channels (approx. 1 Airy unit). DAPI was excited with a 405 nm laser diode, and the emitted fluorescence was filtered through a 420–480 nm band-pass filter. For phalloidin, a 488 nm Argon ion laser was used, and emitted fluorescence was filtered through a 505–530 nm band-pass. Alexa fluor 546 was excited with a 561 nm DPSS laser; the emitted fluorescence was filtered through a 585–690 nm band-pass.

## 3. Results

### 3.1. The isolated Hermes body retains internal flattened cisternae encompassed by a plasma membrane

**Figure 1.**
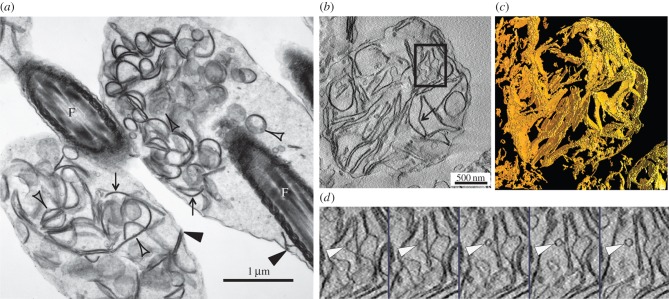
Correspondence of *in situ* and isolated Hermes body by EM tomography. (*a*) EM of *in situ* and (*b*) isolated Hermes body of epididymal sperm of (IS). Flattened cisternae (arrows), vesicular budding (clear arrowheads). Edges of cisternae in proximity to plasma membrane (dense arrowheads). (*c*) Tomography of isolated Hermes body reveals sheet-like unstacked flattened cisternae. (*d*) Series of successive slices (approx. 2.8 nm) of the enclosed area of figure 1*b* revealing a vesicle adjacent to a cisterna. F, flagellum.

Electron microscopy revealed an identical morphology of the Hermes body of epididymal sperm *in situ* (figure 1*a*) to that of its isolated counterpart (figure 1*b*). A plasma membrane surrounded both, and each contained, as the near exclusive organelle, numerous flattened, straight and curved cisternal elements segregated to one pole, but not forming a stacked configuration. Dilated membranous profiles of different shapes and sizes, as well as numerous 50–60 nm spherical vesicles, were also evident (figure 1*a,b*). Tomographical three-dimensional analyses of the isolated Hermes body revealed that the cisternae were flattened and sheet-like in appearance (figure 1*c*). Small vesicles (50–60 nm) were evident (figure 1*d*; electronic supplementary material, SMov.1). Furthermore, close contiguities were often observed between the edges of adjacent cisternae and the plasma membrane (figure 1*a*,*b*). Hence subsequent interrogation of the protein make-up of the Hermes body isolated here should provide insight into the protein make-up of these internal membranes.

### 3.2. Cell map strategy to characterize Hermes body proteins

The Hermes bodies from spermatozoa obtained from proximal regions of adult rat epididymides were characterized by tandem mass spectrometry (electronic supplementary material, table S1). The tandem mass spectra from these samples were combined with those from germ cell-enriched Golgi fractions from adult rat testis homogenates [14] and the resource of tandem mass spectra from endoplasmic reticulum (ER), Golgi and Golgi-derived COPI vesicle proteins from adult rat liver [17] (electronic supplementary material, figure S1). Full-length protein sequences were deduced and assigned to each sample based on the proportion of tandem mass spectra for the protein in each sample. The total number of proteins in the resource is 2922, of which 1151 are found in the Hermes body. For functional relevance, all proteins were allocated into 22 categories (electronic supplementary material, figure S2, derived from table S1) designed for interpreting proteomic data of isolated subcellular fractions of the secretory pathway [17]. As well, electronic supplementary material, table S2 indicates all proteins of the Hermes body in order of their abundance.

Hierarchical clustering of the data of electronic supplementary material, table S1 readily revealed abundant proteins that were enriched or unique to the isolated Hermes bodies, or in common with those in testis Golgi fractions and diminished in liver ER and Golgi fractions (electronic supplementary material, figure S3A). Subsequent selection of proteins for detailed LM-IHC and -IF localizations are indicated in electronic supplementary material, table S3 with the protein distributions in the isolated fractions shown in electronic supplementary material, figure S3B, and the proteins localized by IHC, IF or immuno-EM indicated in electronic supplementary material, figure S3C in comparison with those proteins whose localization was already studied in [14].

### 3.3. Concentration and localization of TMED/p24 membrane trafficking proteins in flattened cisternae of Hermes bodies supports a Golgi identity

**Figure 2.**
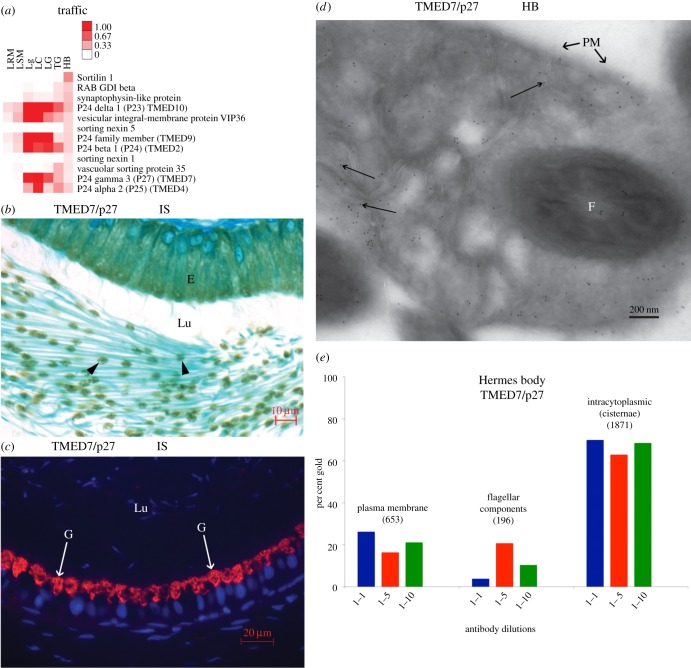
Hermes bodies localization of TMED7/p27 by LM and EM *in situ*. (*a*) Heat map of the 12 most abundant proteins of the Traffic category. (*b*) IHC shows the localization of TMED7/p27 in epididymal Hermes bodies. (*c*) IF reveals Golgi reactivity of epididymal epithelial cells for TMED7/p27. However, germ cell immunoreactivity is not seen due to the low sensitivity of the IF protocol. (*d*) EM immunolocalization of TMED7/p27. Gold particles (arrows) over flattened cisternae, plasma membrane and flagella of sperm. (*e*) Gold particle quantification in cryosections of Hermes body of sperm. The total number of gold particles scored over the plasma membrane, intracytoplasmic (cisternae) or flagella are indicated above each histogram representing different dilutions of anti-TMED7/p27. E, epithelium; F, flagellum; G, Golgi reactivity; HB, Hermes body; IS, initial segment; Lu, lumen; PM, plasma membrane.

Several TMED (p24 family) proteins for membrane traffic (figure 2*a*) were concentrated in isolated Hermes bodies. TMED7/p27 is Golgi restricted and expressed at all steps of male germ cell differentiation in the testis [14]. In epididymis, the protein shows a Hermes body segregation in sperm (figure 2*b*) and Golgi localization in epithelial epididymal cells (figure 2*c*). By EM immunogold labelling, TMED7/p27 was concentrated in flattened cisternae of the Hermes body (figure 2*d*) of epididymal sperm *in situ*. Quantification indicated 67 ± 4% gold particles (mean ± s.d., *n* = 3) over the flattened cisternae, 21 ± 5% on the plasma membrane of the Hermes body and 12 ± 9% considered as non-specific over flagellar components (figure 2*e*; electronic supplementary material, table S4). Negligible gold particles were seen without primary antibody. Hence, TMED7/p27 is characterized as a Golgi protein localized to the internal membranes of the Hermes body of epididymal sperm representing the first documentation of this protein to the structure and confirming a Golgi identity [9].

### 3.4. Protein abundance assigns a major function in glucose uptake and glycolysis

**Figure 3.**
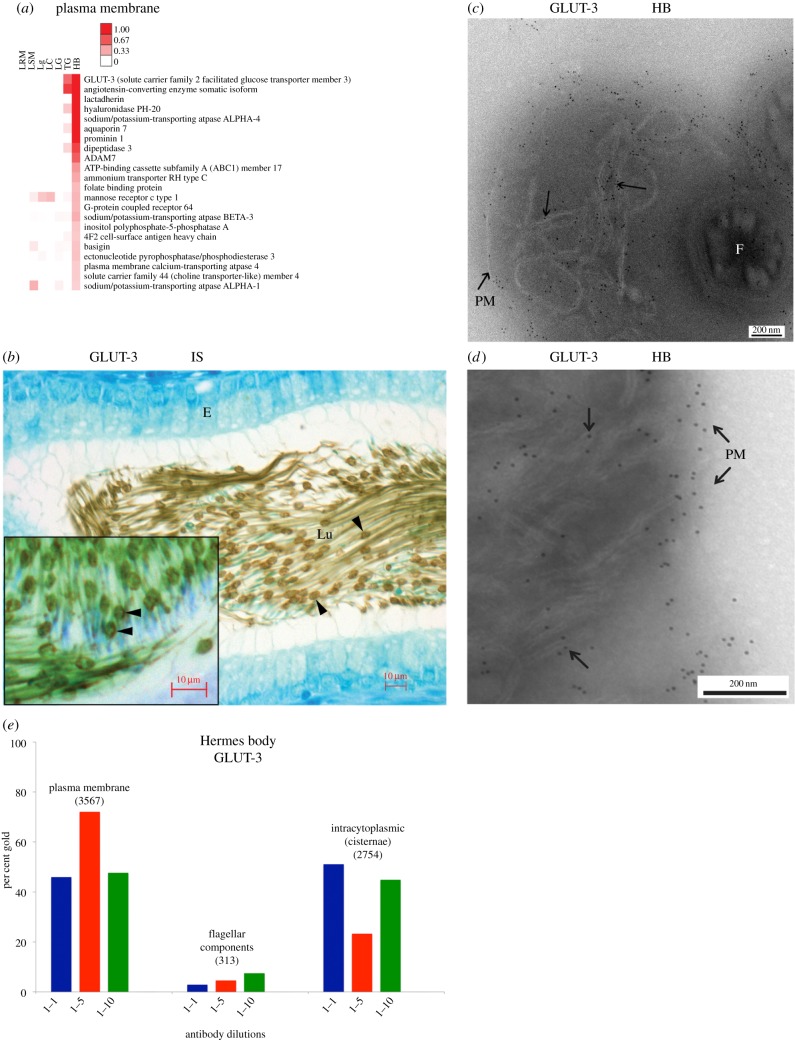
*In situ* localization of GLUT-3. (*a*) Heat map of 22 proteins in plasma membrane category (ordered by abundance and averaged for each subcellular fraction). (*b*) LM-IHC of GLUT-3 in Hermes body of epididymal sperm and (inset) reaction restricted to one pole. (*c*,*d*) EM cryosections of Hermes body of sperm in IS immunolabelled with anti-GLUT-3. Gold particles associated with PM and flattened cisternae. (*e*) Gold particle labelling at primary antibody dilutions indicated was scored over the plasma membrane, intracytoplasmic (cisternae) or flagellar components as shown in the electronic supplementary material, table S4. The total number of gold particles scored is indicated above each histogram. Abbreviations as in figure 2.

The most abundant protein of the isolated Hermes body (electronic supplementary material, table S2) was the high-affinity GLUT-3. Categorized to plasma membranes (figure 3*a*), GLUT-3 is a known sperm flagellar plasma membrane protein, identical to neuronal GLUT-3 [30]. Other known sperm plasma membrane proteins detected in the isolated Hermes body included aquaporin 7 [27], angiotensin-converting enzyme [31,32], PH20 [33], dipeptidase 3 [34], mannose receptor [35], ABC1 member 17 [36], folate binding protein [37] and the highly abundant sodium potassium ATPase subunit alpha 4, a germ cell-derived known sperm plasma membrane protein, essential for motility and fertility [38,39]. ADAM 7 was prominent, as were 11 other family members (electronic supplementary material, table S2) all known to be sperm associated [40–42]. Several of these proteins have been characterized in proteomic studies of sperm [43–45]. These were not seen by proteomics of isolated droplets by Yuan *et al.* [11] due to their use of two-dimensional gels that cannot resolve membrane proteins.

LM-IHC for GLUT-3 revealed localizations in small focal elliptical dilations defined as the Hermes bodies (figure 3*b*), with immunoreactivity concentrated to one pole (figure 3*b*, inset). Although less concentrated, immunoreactivity was also observed along the entire midpiece of the sperm flagellum (figure 3*b*). Immunogold labelling of GLUT-3 on ultrathin cryosections of *in situ* initial segment epididymal tissue revealed a major concentration of gold particles over the plasma membrane of the Hermes body; gold label is also concentrated over the internal flattened cisternae (figure 3*c*,*d*). As shown in figure 3*e* and electronic supplementary material, table S4, 55 ± 15% (mean ± s.d., *n* = 3) of all gold particles are on the plasma membrane, with intracellular cisternal GLUT-3 labelling at 40 ± 15% and non-specific gold labelling to flagellar components at 5 ± 2%. Negligible gold particles are found in sections without primary antibody (electronic supplementary material, table S4). The localization of a subset of GLUT-3 to the same internal membranes as TMED7/p27 is consistent with a membrane trafficking function for the internal membranes.

**Figure 4.**
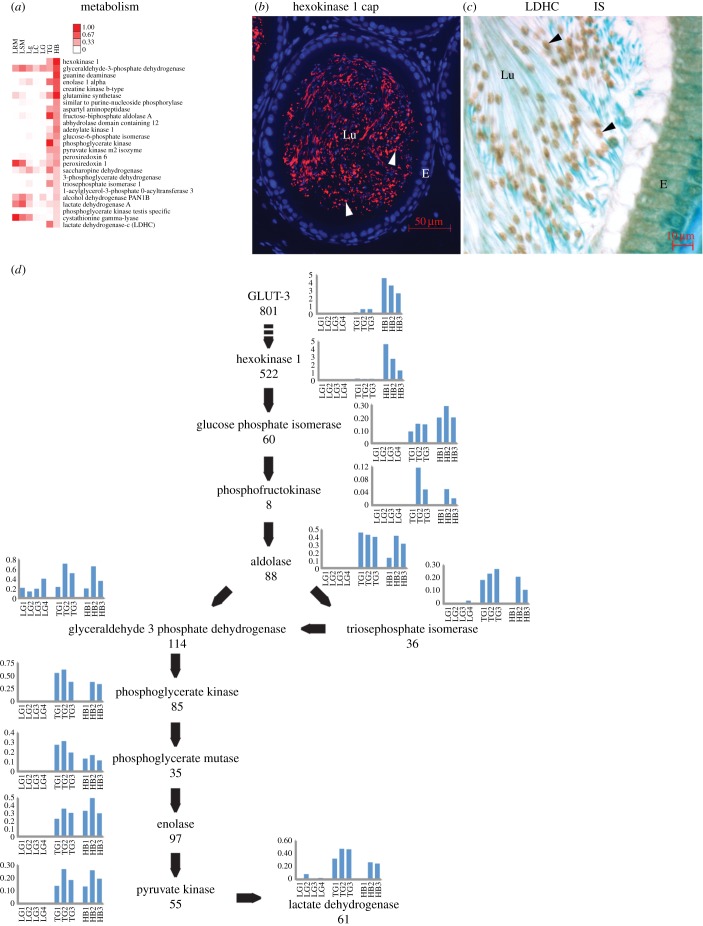
Hermes body and glycolysis as deduced from protein abundances and localizations. (*a*) Twenty-five proteins in metabolism category. Scale indicates percentage total peptides. (*b*) IF of Hexokinase 1 and (*c*) IHC of lactate dehydrogenase C. Both show concentration in Hermes bodies of sperm in lumina of the IS. (*d*) Proteomics of the glycolytic pathway. Histograms represent peptide abundances in separate isolates for the indicated proteins. Total number of peptides (*n* = 3) characterized in the isolated Hermes bodies is indicated below each protein name. LG, liver Golgi; TG, testis Golgi. Other abbreviations as in figure 2.

The second most abundant protein of the isolated Hermes body is the first enzyme in glycolysis, Hexokinase 1 (electronic supplementary material, table S2). This is the most abundant protein in the metabolism category (figure 4*a*). Hexokinase 1 localizes to the Hermes body of sperm in the epididymal lumen (figure 4*b*). Lactate dehydrogenase C the 25th most abundant protein of the category is concentrated largely in the Hermes body (figure 4*c*). All other glycolytic enzymes are characterized by proteomics albeit at markedly different abundances (figure 4*d*). Thus, the Hermes body concentrates GLUT-3 and all glycolytic enzymes, extending the observations of Yuan *et al.* [11], who observed only the soluble glycolytic enzymes by their two-dimensional gels.

### 3.5. Hermes body concentration of filamentous actin and tubulin for migration

Cytoskeletal proteins are highly abundant (electronic supplementary material, figure S4A) with actin gamma 1 as the most abundant. Tubulin beta 5 and tubulin alpha 1C are also prominent, with the latter localized by LM-IHC to the Hermes body (electronic supplementary material, figure S4*b*). Myosins (myosin 1B, myosin VB, myosin ID, myosin IIIB) are enriched as well as other prominent cytoskeletal proteins (electronic supplementary material, figure S4*a*). Several have been localized previously to sperm. These include spectrin [46], BASP1 (NAP22) [47] and the motility-enhancing protein gelsolin [48]. Controls for all LM-IHC or LM-IF using non-immune serum or the absence of primary antibody revealed no immunoreactivity over tissue sections (electronic supplementary material, figure S4C).

**Figure 5.**
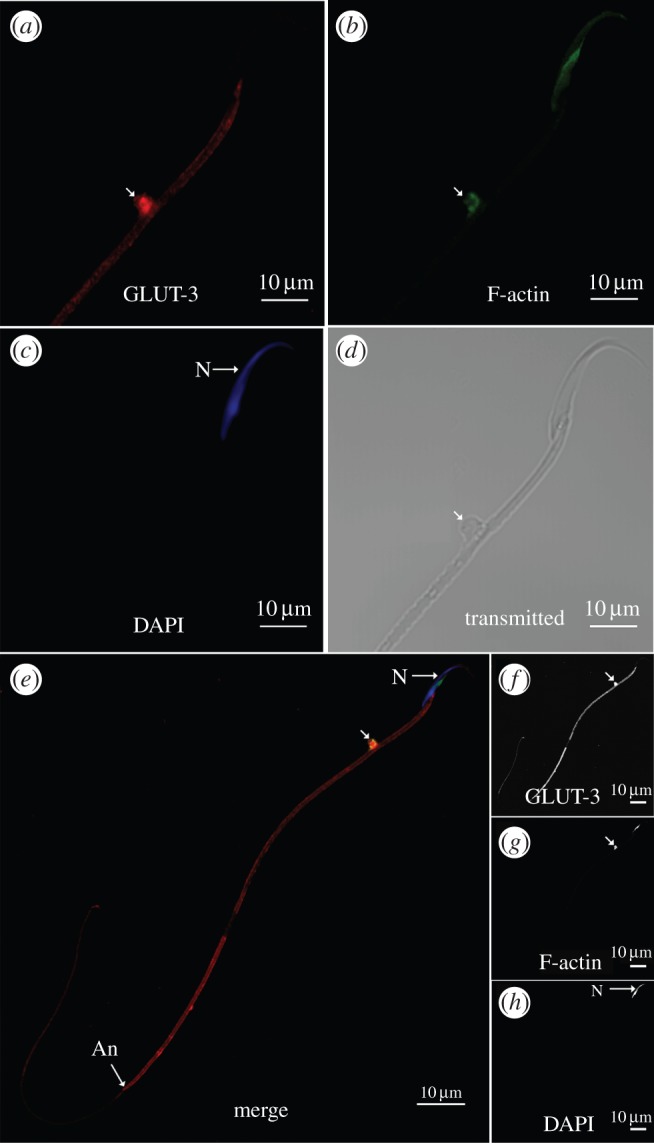
Colocalization of GLUT-3 and filamentous actin in the Hermes bodies of isolated epididymal sperm. (*a*) Visualization of GLUT-3 and (*b*) phalloidin (F-actin) in Hermes body. (*c*) Nucleus (N) stained with DAPI. (*d*) Sperm head and flagellum in transmitted light. Scale bars = 5 µm. (*e*) Reactive Hermes body (arrow) with GLUT-3 (red), phalloidin (green) and DAPI (blue) channels merged. Annulus (An). Depicted in black and white are individual reactions for GLUT-3 (*f*), phalloidin (*g*) and DAPI (*h*). Scale bars, 10 µm.

Phalloidin staining for filamentous actin visualized its co-localization along with GLUT-3 to the Hermes body (figure 5*a–c*), as also depicted in transmitted light (figure 5*d*), merged images (figure 5*e*), and black and white representations (figure 5*f*–*h*). This is the first observation of filamentous actin concentrated in the Hermes body of epididymal sperm.

### 3.6. Extensive membrane trafficking and fusion constituents in Hermes bodies as deduced from abundant COP coats, Clathrin, SNAREs, annexins, AAA+ proteins and trafficking GTPases

**Figure 6.**
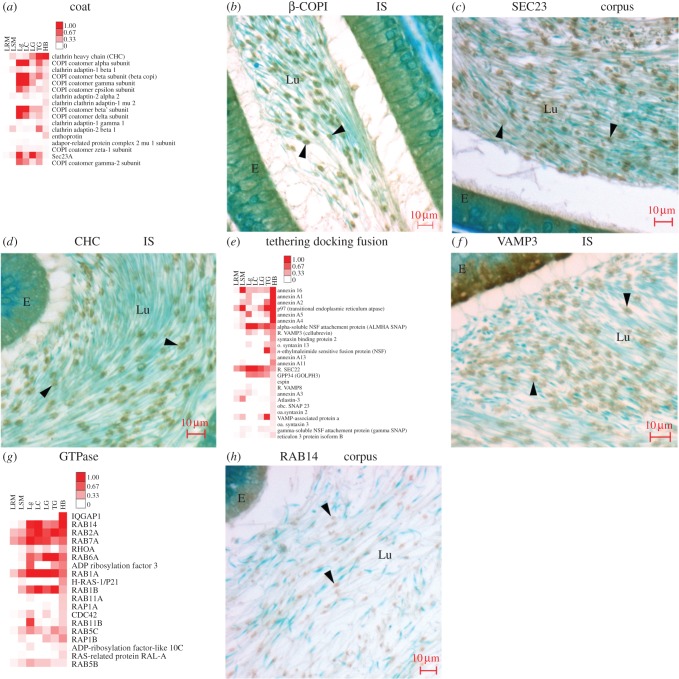
Coated vesicle, membrane fusion and Rab proteins in Hermes bodies. (*a*) Heat map of 17 proteins in coat category sorted by their abundance in Hermes bodies. (*b*–*d*) Localization of different coat proteins as indicated for each. (*e*) Heat map of the most abundant 25 proteins of tethering, docking and fusion sorted by their abundance in Hermes bodies. (*f*) VAMP3 localization to Hermes bodies. (*g*) Heat map of the most abundant 19 proteins of GTPase category sorted by their abundance in Hermes bodies. (*h*) RAB14 localization in Hermes bodies. Abbreviations as in figure 2.

COPI and COPII coatomer, clathrin heavy chain and the clathrin adaptins AP1 and AP2 are abundant, as seen in the Coat category (figure 6*a*). All seven subunits of COPI coatomer of Golgi retrograde directed COPI vesicles [49] are characterized (figure 6*a*), with β-COP visualized (figure 6*b*). The anterograde trafficking ER to Golgi vesicles as represented by COPII coatomer subunits are lower in abundance (figure 6*a*) but readily localized (e.g. Sec23, figure 6*c*), as were clathrin coat constituents representing clathrin-coated vesicles (e.g. clathrin heavy chain, figure 6*d*) and its Golgi-restricted adaptor protein AP1 and endocytic-restricted AP2 (not shown). A localization of clathrin heavy chain to Hermes bodies of spermatozoa confirms an earlier study [50].

Abundant SNAREs are characterized in the tethering, docking and fusion category (figure 6*e*; e.g. VAMP3, figure 6*f*). Annexins are highly abundant, as are the AAA proteins p97 (see below), NSF and its associated alpha SNAP (figure 6*e*). The atlastins and reticulons, known to be involved in membrane curvature, tubulation and fusion [51], are also abundant in the Hermes body (figure 6*e*).

The most abundant GTPase is IQGAP (figure 6*g*), known to coordinate actin polymerization and microtubule formation [52] as well as signalling [53]. The GTPase category includes abundant Rab trafficking proteins. The most abundant Rab is Rab14 (figure 6*g*), known to be involved in the trafficking and internalization of the GLUT-3 paralogue, GLUT-4, in other cell types [54,55]. Rab14 concentrates in the Hermes body of epididymal sperm *in situ* (figure 6*h*). The Golgi marker Rab6a was the sixth most abundant GTPase.

### 3.7. ER, ribosomal and elongation factor proteins are abundant

**Figure 7.**
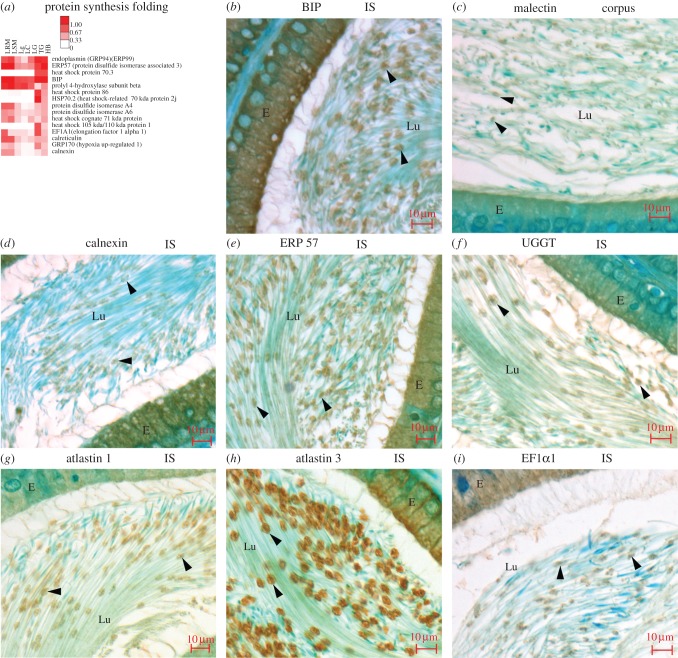
ER proteins are concentrated in isolated Hermes bodies and localized to Hermes bodies *in situ*. (*a*) Most abundant 15 proteins of protein synthesis and folding category. (*b*–*i*) Localization of abundant ER proteins as indicated. Abbreviations as in figure 2.

Categorized in the protein synthesis and folding category (figure 7*a*), several ER proteins concentrate in the Hermes body including BIP, malectin, calnexin, ERP57 and UGGT (figure 7*b*–*f*). Proteins involved in ER membrane curvature (reticulons 1 and 3) and fusion (atlastins 1, 3) (figure 7*g–h*) are also abundant, as indicated in electronic supplementary material, table S2. This is the first localization of these proteins to the Hermes body. As expected from the prior studies of Chauvin *et al.* [44] on whole epididymal sperm, 68 ribosomal proteins are characterized, as well as the ribosome-associated translation elongation factors EF1A1 and EF2 (electronic supplementary material, table S2), with EF1A2 localized *in situ* (figure 7*i*). These data support the hypothesis of Chauvin *et al.* [44] of an intact translation apparatus in sperm, which we localize to the Hermes body.

### 3.8. Abundant secretory proteins from Sertoli cells and epithelial lining cells are enriched on Hermes bodies

By proteomics, abundant secretory proteins are observed in the isolated Hermes body (categorized under biosynthetic cargo; electronic supplementary material, figure S5). Albumin, transferrin and ceruloplasmin are highly abundant, but these have previously been considered as serum-derived contaminants of epididymal spermatozoa [44]. Such contamination is ruled out by our proteomic data. ApoE, complement C3 and hemopexin are diminished in the Hermes bodies as compared with liver organelles. These proteins are exclusively made in liver, while albumin and transferrin are biosynthesized in Sertoli cells [56,57]. As expected, albumin and transferrin are localized to the Hermes body *in situ* (electronic supplementary material, figure S5*b*–*c*) as well as in the Sertoli cell of the testis where they are synthesized (electronic supplementary material, figure S5*d*). Their selective concentration is consequent with their adsorption as Sertoli cell-secreted proteins. Albumin is needed for cholesterol removal and the acquisition of fertility [58,59]. Transferrin and ceruloplasmin are needed for iron uptake [60] as an essential feature for all living cells.

Other proteins (electronic supplementary material, figure S5*a*), derived from epithelial epididymal cells, also associate with the sperm surface and are seen in the isolated Hermes body fractions (e.g. cysteine-rich secretory protein 1 [61], epididymal-expressed lipocalin-5 [62] and clusterin [63]).

### 3.9. The Hermes body compartmentalizes stress proteins

By proteomics, several antioxidant enzymes, especially glutathione S transferases (GSTs), are abundant and classified in the detoxification category (electronic supplementary material, figure S6*a*). Several of these have been localized previously to cytoplasmic droplets [64,65]. However, we can add 18 different heat shock proteins as protective against stress (characterized in the protein synthesis and folding category; figure 7*a*). This includes HSP70.3 and HSP86 (figure 7*a*), and the germ cell-specific HSP70.2 protein (localized in electronic supplementary material, figure S6*b*,*c*). The HSP70 ER luminal protein, GRP170 (localized in electronic supplementary material, figure S6*d*), is abundant (figure 7*a*). The peroxiredoxins 1, 3 and 6, of sperm [66], scavenge reactive oxygen species and are classified in the Metabolism category (figure 4*a*; localization of peroxiredoxin1 in electronic supplementary material, figure S6*e* and peroxiredoxin 6 in figure S6*f*). Our work is the first to reveal a Hermes body localization for several of these proteins that provide protection of whole sperm to oxidative stress [67–69].

### 3.10. Protein loss in Hermes bodies during sperm transit

Differential protein loss along the epididymal duct is represented by a comparison of two members of the atlastin family of membrane fusion proteins that are evident in the isolated Hermes body (electronic supplementary material, table S2). The gene product expressed in all cells, atlastin 3 [70], and prominent in the Hermes body (figure 6*e*), is compared with atlastin 1, which is expressed mainly in testis and brain [71]. Atlastin 3 is present throughout the epididymal duct including the cauda region (electronic supplementary material, figure S7*a*); atlastin 1 is evident in the Hermes body of sperm transiting proximal regions of the duct (electronic supplementary material, figure S7*b*) but absent from the cauda region (electronic supplementary material, figure S7*c*). Hence the Hermes body initially expresses both atlastins, but only the ubiquitous atlastin 3 remains in sperm after epididymal release.

As shown in electronic supplementary material, figure S7*d*, 12 proteins are lost during sperm transit through the epididymis; eight in the caput and four more in the corpus. Consistent with their loss by degradation is the finding of an enrichment of the Hermes body in proteasome constituents and ubiquitin conjugating enzymes categorized in the proteasome category (electronic supplementary material, figure S7*e*). The most abundant is the known germ cell ubiquitin ligase cullin3 [72], also known to regulate the structure of Golgi apparatus in somatic cells [73]. All ATP-dependent protein degradation constituents, including P97, the 20S proteasome and ubiquitin, are seen in Hermes bodies throughout the epididymal duct (electronic supplementary material, figure S7*f*–*h*). Besides revealing the concentration of the regulatory and structural proteins for ATP-dependent proteasome degradation in the Hermes bodies, the selective loss of 12 of the 43 Hermes body proteins during sperm transit in the epididymis supports the hypothesis that ATP-dependent protein degradation in the Hermes body is required for sperm maturation [74,75].

### 3.11. Hermes body in late step 19 spermatids

**Figure 8.**
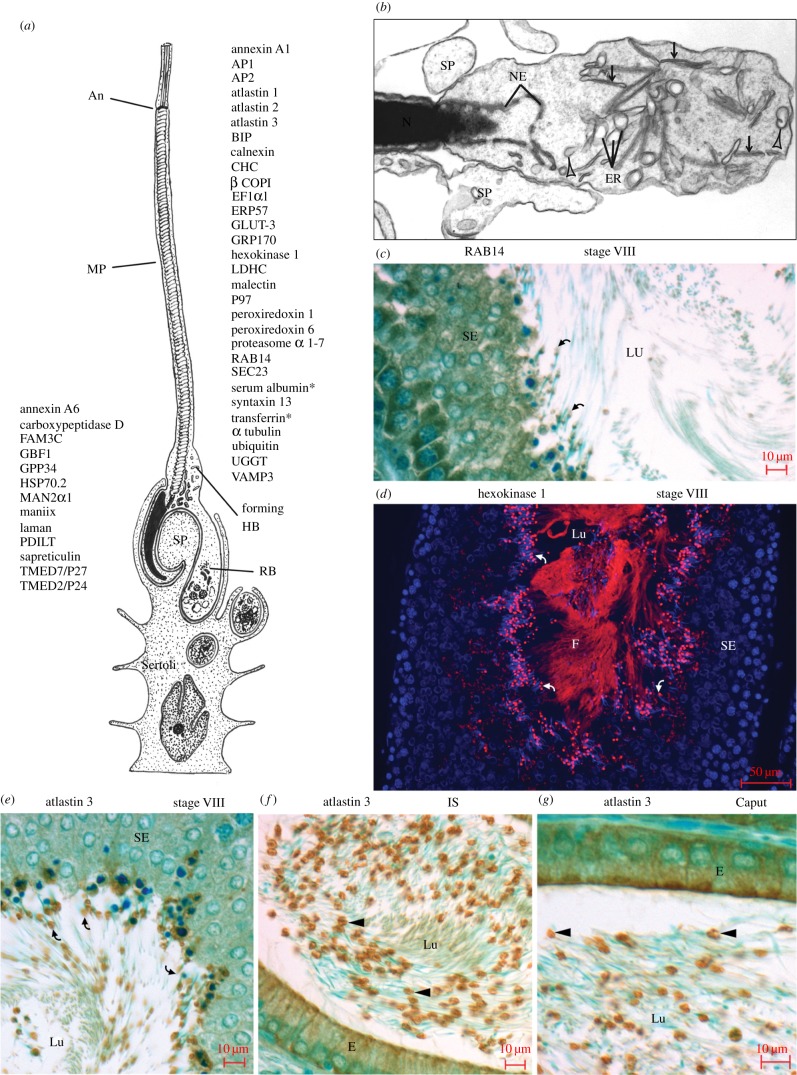
The Hermes body is formed in the step 19 spermatid. (*a*) Schematic of step 19 spermatid with the flagellar midpiece (MP) ending at the annulus (An) embedded in Sertoli cell processes (SP) that phagocytose excess cytoplasm and organelles into the residual body (RB). Thirteen Golgi-localized proteins of germ cells are indicated on left and 30 non-Golgi proteins on right. All 43 proteins are immunoreactive in step 19 spermatids. Asterisks: Sertoli cell derived albumin and transferrin. (*b*) EM of step 19 spermatid enveloped by Sertoli cell processes (SP). The forming Hermes body reveals flattened cisternae (arrow), distended membranous elements (DE) and coated vesicles (arrowheads); NE, nuclear envelope; N, nucleus. The forming Hermes bodies (curved arrows) of step 19 spermatids are reactive as noted for Rab14 by IHC (*c*) and Hexokinase 1 by IF (*d*) in the SE. (*e*) Atlastin 3 concentration in the forming Hermes body of step 19 spermatids. (*f*) Prominent Atlastin 3 immunoreactivity in Hermes bodies of sperm in the IS. (*g*) Atlastin 3 in Hermes bodies of sperm of the caput epididymidis. Abbreviations as in figure 2.

The Hermes body forms in late step 19 spermatids as a segregated bulge at the neck or connecting piece of the flagellum (figure 8*a*), enveloped by Sertoli cell processes (figure 8*b*). Flattened and dilated cisternae are concentrated in the structure, as are vesicles of different sizes (figure 8*b*).

The hypothesis that the step 19 spermatid concentrated all proteins of the Hermes body of epididymal sperm was tested here by looking at all 43 proteins localized to the Hermes body *in situ.* Thirteen of these represent Golgi-derived proteins, already demonstrated to be concentrated in the step 19 spermatid by us [14], and summarized on the left of figure 8*a*. The remaining 30 proteins of Hermes bodies were all localized to the step 19 spermatid in this study (summarized on the right side of figure 8*a*). Examples are shown for Rab14 (figure 8*c*) and Hexokinase 1 (figure 8*d*). As illustrated in figure 8*e*–*g*, the abundant smooth ER fusion protein atlastin 3 of the forming Hermes body in step 19 spermatids is compared with that of the Hermes body in epididymal sperm.

**Figure 9.**
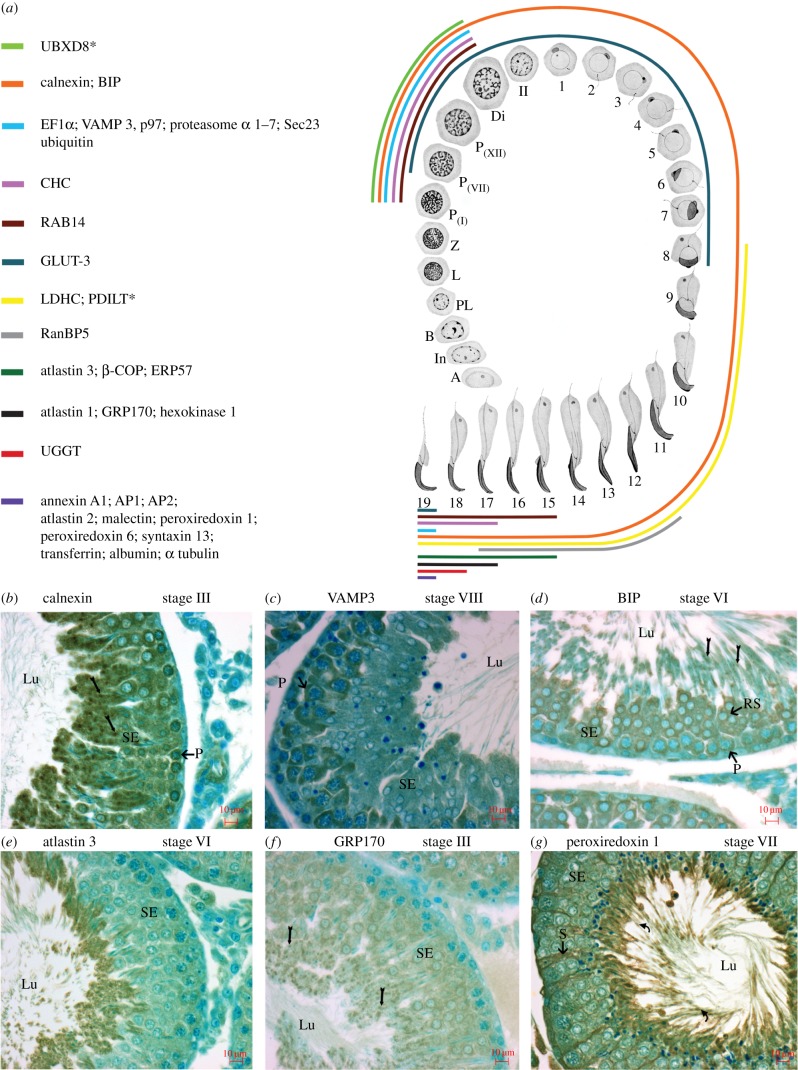
Selective protein expression of Hermes body proteins during spermatogenesis. (*a*) Schematic illustrating distribution of non-Golgi-localized proteins in germ cells during spermatogenesis. Each coloured line represents the expression of immunoreactivity for a non-Golgi-localized protein or combination of proteins in germ cells as they differentiate in the SE of the testis. Spermatogonia (types A, intermediate (In) and B). Primary spermatocytes: preleptotene (PL), leptotene (L), zygotene (Z), pachytene (P), diplotene (Di) and secondary spermatocytes (II), and steps 1–19 of spermiogenesis (modified from [76]). PDILT (asterisk) is also noted as a Golgi-localized protein, as is UBXD8 (asterisk) as previously documented [14]. LM-IHC of the SE of testis immunostained with anti-calnexin (*b*), Vamp3 (*c*), BIP (*d*), atlastin 3 (*e*), GRP170 (*f*) and peroxiredoxin 1 (*g*). Code for labels showing reactivity: pachytene spermatocytes (P), small clumps representative of ER in late spermatids (arrows), forming Hermes bodies (curved arrows), round spermatids (RS), cytoplasmic of late spermatids (asterisks), Sertoli cells (S). Abbreviations as in figure 2.

The differential expression of the 30 non-Golgi proteins shown in figure 8*a* was deduced here for all germ cells during differentiation in the testis (figure 9*a*), with representative examples shown in figure 9*b*–*g*. Taken together with the differential expression of the 13 Golgi proteins previously documented [14], the hypothesis that the step 19 spermatid is the site of Hermes body biogenesis for epididymal sperm is supported at the molecular protein level as well as previously at the morphological level [9].

## 4. Discussion

### 4.1. The Hermes body concentrates membrane trafficking proteins, GLUT-3, glycolytic enzymes, filamentous actin and tubulin alpha 1C

This is the first report of abundant membrane trafficking constituents in epididymal sperm here sequestered to the Hermes body. A Golgi apparatus contribution from the TMED proteins is exemplified by TMED7/p27, which localized by EM to the internal flattened cisternae, as did about one-third of GLUT-3, the major plasma membrane protein of epididymal sperm. TMED7/p27 is required to transport plasma membrane cargo from the ER through the Golgi to the plasma membrane [77]. Five TMED paralogues were of high abundance in the Hermes body and together may regulate GLUT-3 expression at the surface as the rate-limiting step in glucose uptake, glycolysis and therefore the initiation of sperm motility.

Besides Golgi contributions, the internal membranes of the Hermes body were deduced as endosomal with clathrin, AP2, VAMP3 and Rab14, indicative of endocytic membrane trafficking [78–80]. Traffic from the ER to the Golgi is exemplified by all constituents of the calnexin cycle, BiP as well as COPII coats for transport to the Golgi. Golgi adaptin AP1 for clathrin-coated vesicles and all COPI coat subunits for Golgi-mediated anterograde COPI vesicle trafficking for intraGolgi and Golgi to ER retrograde traffic are represented, and thus constitute a complete secretory pathway. Also represented are the atlastins and reticulons for ER membrane curvature and fusion.

During epididymal sperm maturation, ATP generation from glycolysis directs the initiation of sperm motility as confined to the principal piece of the sperm flagellum [81–83]. Previous proteomics studies on sperm (including epididymal sperm) [41–43,82] uncovered GLUT-3 and all glycolytic metabolic enzymes as prominent. In humans, 436 proteins of the 1056 found in sperm belong to various metabolic pathways, with glycolytic proteins the highest expressed [84]. The high concentration and localization here of GLUT-3 and glycolytic enzymes to the Hermes body indicates that it is also in this macrostructure that glycolysis generates the ATP needed to initiate sperm motility, and this involves the internal flattened membranes contained therein.

Also shown here for the first time is the sequestration of filamentous actin and the localization of tubulin alpha 1C to the Hermes body *in situ* in the proximal epididymis. In this region, the Hermes body undergoes a dynamic migration along the flagellar midpiece. This event coincides with the acquisition of sperm motility [5,6,54]. Migration of the Hermes body is coincident with the movement of the internal flattened cisternae in a spiral manner while retaining a close association with the sperm plasma membrane [85]. Prior to the localization of filamentous actin and tubulin alpha 1C here, nothing was known of the cytoskeletal protein constituents that may regulate this migration.

The Hermes body in humans is known to be linked to fertilization [6]. However, the functional significance of the proteins characterized and localized here will require detailed analysis on a protein by protein basis.

### 4.2. The Hermes body and protein degradation

A complete ATP-dependent protein degradation machinery is concentrated in the Hermes body. This includes ubiquitin, all subunits of the proteasome, P97 and highly abundant cullin 3, the sperm ubiquitin ligase also protective for stress [72,86]. Selective protein loss is shown for 12 proteins of the Hermes body as sperm transit for 10 days from the initial segment to the cauda epididymidis [87]. Proteasome subunits and ubiquitin conjugating enzymes have also been characterized recently in a proteomics study of whole epididymal sperm [44] and localized *in situ* [88]. Ijiri *et al*. [89] also observed a loss of proteins during sperm passage through the epididymis. Strikingly, they concluded that HSP70.2 was present in the Hermes body of the caput sperm, but absent from those of the cauda epididymidis exactly as found here. Although Ijiri *et al*. [89] attributed this to loss of the Hermes body itself, we concluded through the more extensive markers analysed here that the Hermes body is maintained in the cauda epididymidis, although whether or not they are attached to sperm is debatable [2,5]. For humans, the Hermes body appears to be maintained in association with sperm after release from the epididymis [6].

Regardless, selective protein loss through ATP-dependent degradation would modify sperm as they traverse the epididymal duct. Taken together with the proteins of the biosynthetic secretory apparatus characterized here and by Chauvin *et al*. [44], far from being a cell devoid of a translation machinery, epididymal sperm may be reassessed as in a dynamic state of protein turnover as proposed for all cells [90].

### 4.3. Hermes body biogenesis at step 19 of spermiogenesis

Transcription ceases immediately after acrosome formation with protein expression thereafter under translational regulation [91]. The translation controlled pathway for organelle modification and morphogenesis of germ cells [92] must be prominent at step 19 to encompass subsets of plasma membrane, cytosolic, Golgi, ER and endosome proteins within the Hermes body of epididymal spermatozoa. That some of these components are merely remnants of the phagocytosis of excess germ cell cytoplasm by Sertoli cells in step 19 spermatids cannot be formally ruled out, but will be resolved as more functional studies ensue.

The work here provides a spatial context for protein concentration in an apparently unique structure designed in epididymal sperm, the Hermes body.

## Supplementary Material

HermesSuppFigure-1.tif

## Supplementary Material

HermesSuppFigure-2.tif

## Supplementary Material

HermesSuppFigure-3.tif

## Supplementary Material

HermesSuppFigure-4.tif

## Supplementary Material

HermesSuppFigure-5.tif

## Supplementary Material

HermesSuppFigure-6.tif

## Supplementary Material

HermesSuppFigure-7.tif

## Supplementary Material

Supplemental Table 1 (S1). Supplemental Table 2 (S2). Supplemental Table 3 (S3). Supplemental Table 4 (S4).
