# Supplementary figures and images for: Compartmentalization of membrane trafficking, glucose transport, glycolysis, actin, tubulin and the proteasome in the cytoplasmic droplet/Hermes body of epididymal sperm
Source: Open Biol. 2015 Aug 26;5(8):150080. doi: 10.1098/rsob.150080 (PMC4554921; doi:10.1098/rsob.150080)

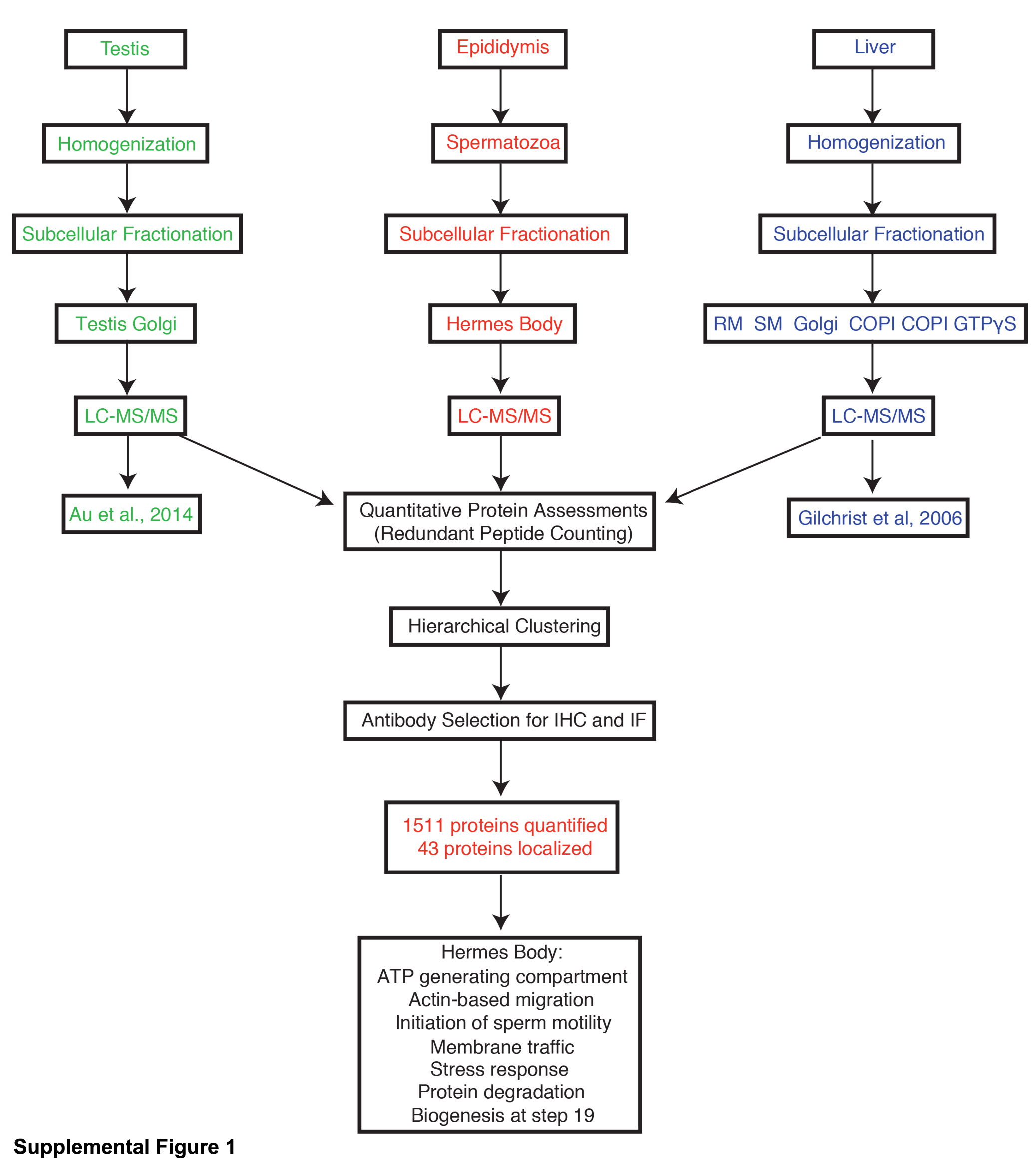

Supplement: HermesSuppFigure-1.tif [file rsob150080supp1.tif]

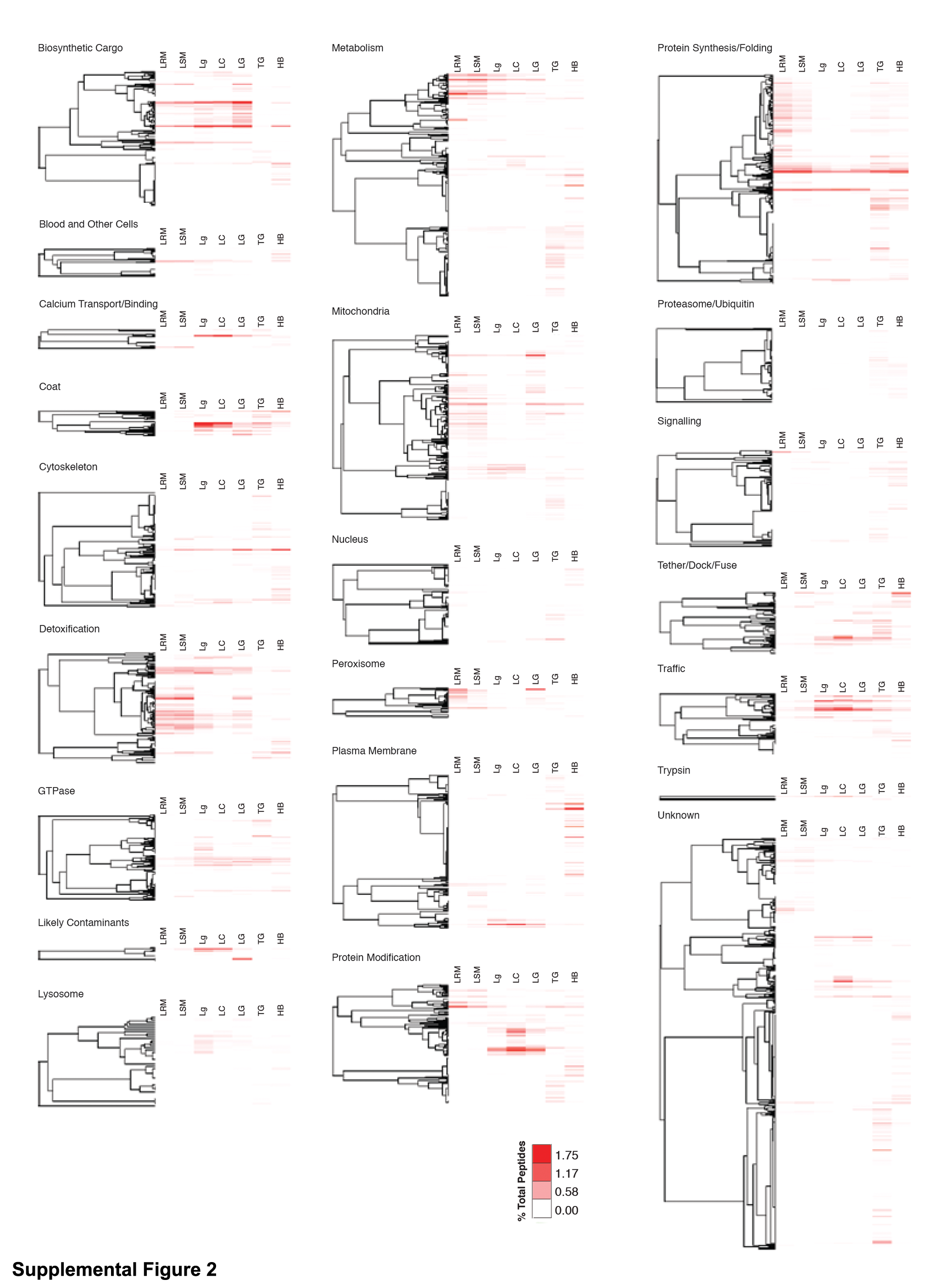

Supplement: HermesSuppFigure-2.tif [file rsob150080supp2.tif]

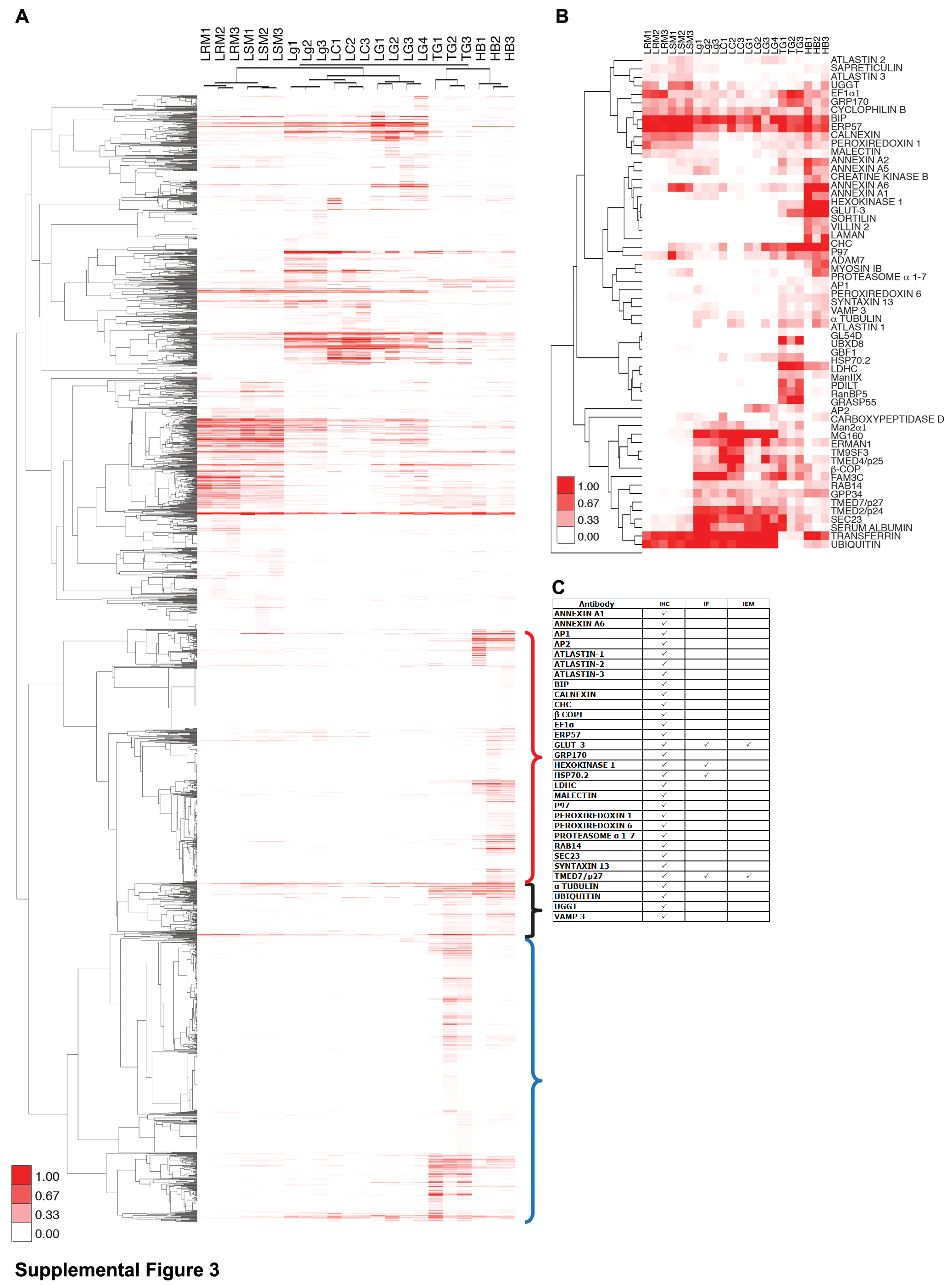

Supplement: HermesSuppFigure-3.tif [file rsob150080supp3.tif]

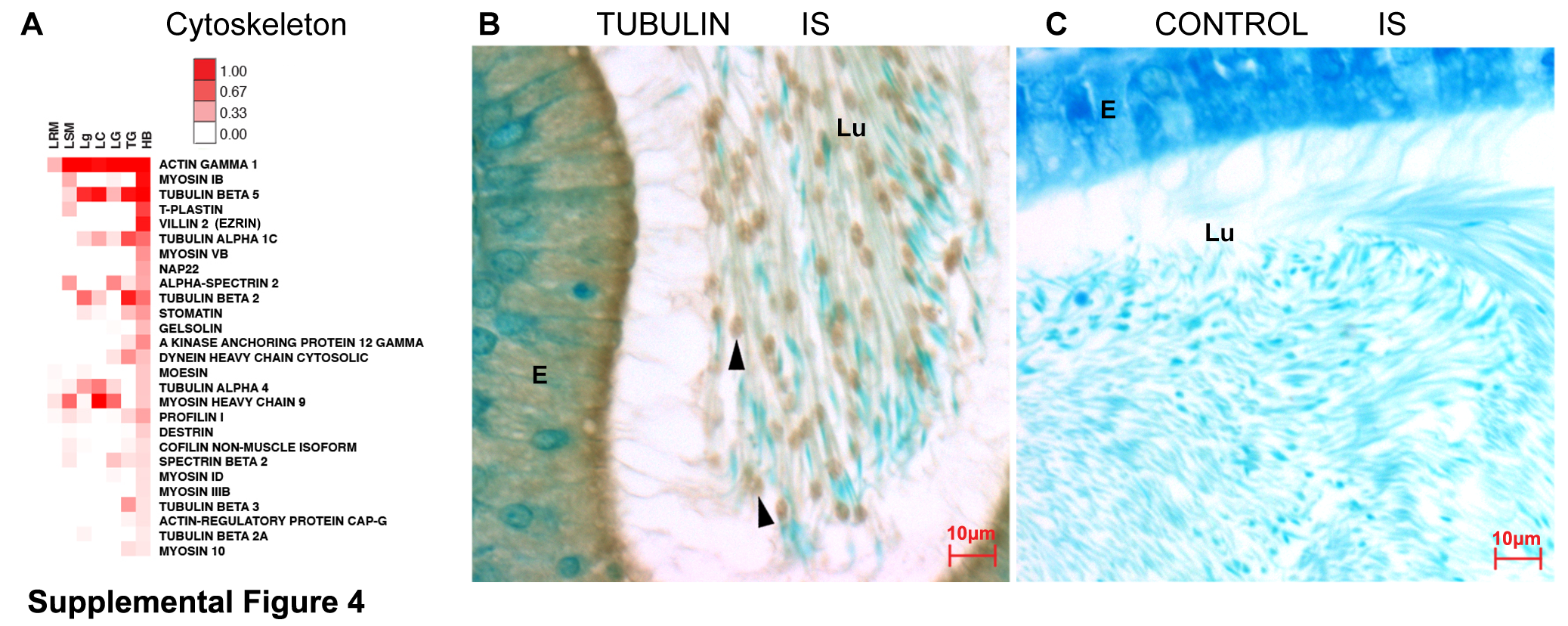

Supplement: HermesSuppFigure-4.tif [file rsob150080supp4.tif]

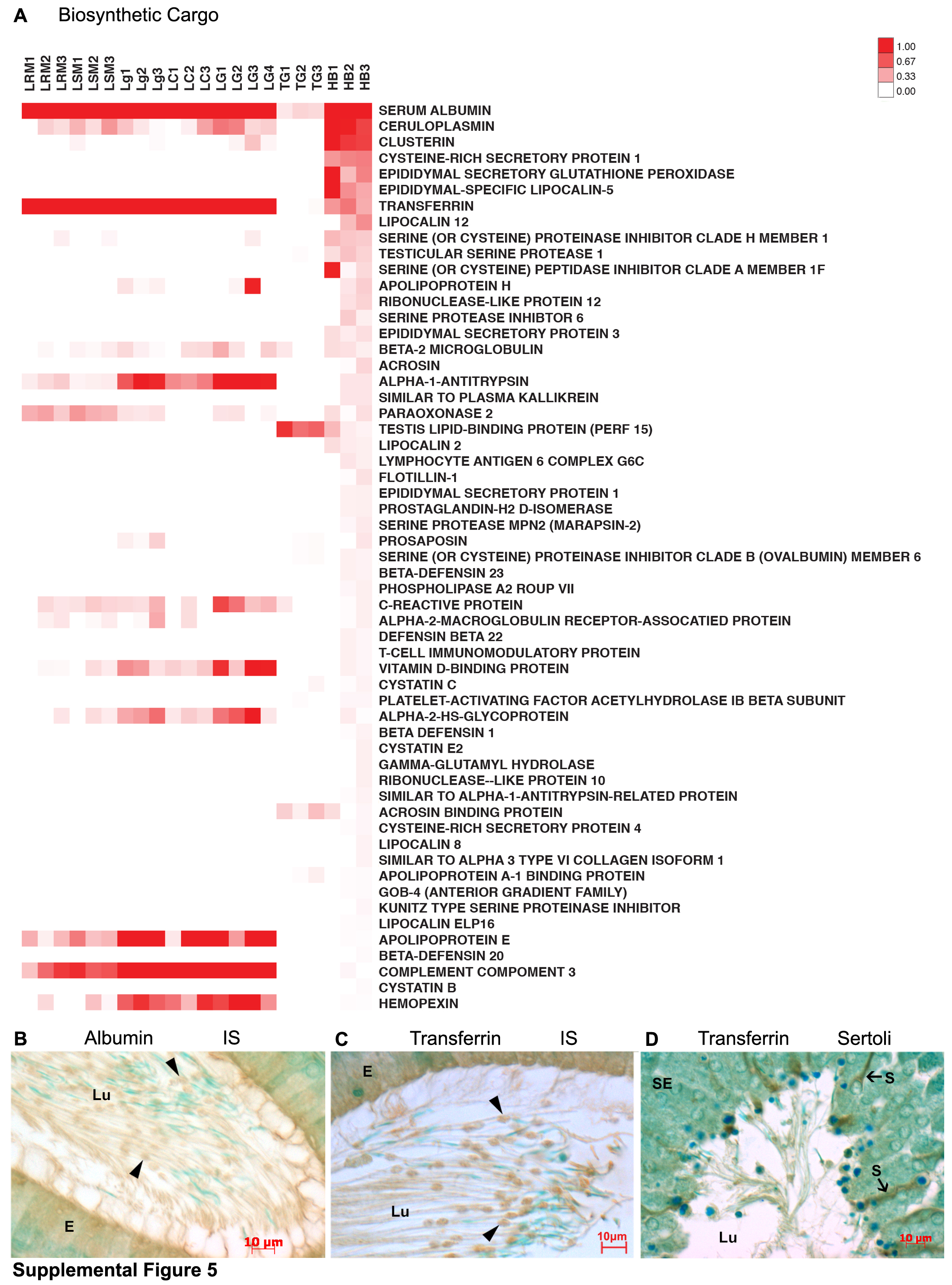

Supplement: HermesSuppFigure-5.tif [file rsob150080supp5.tif]

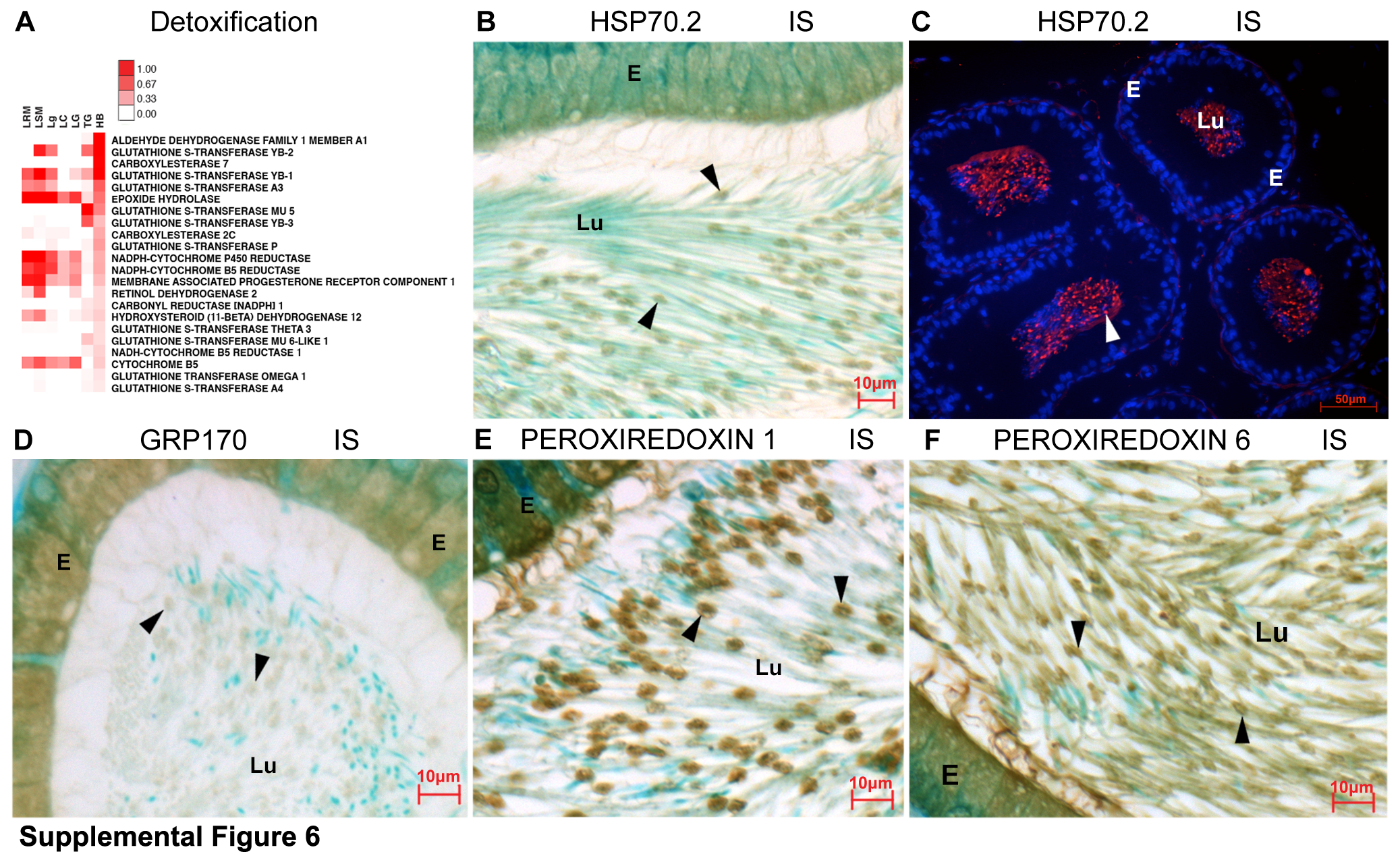

Supplement: HermesSuppFigure-6.tif [file rsob150080supp6.tif]

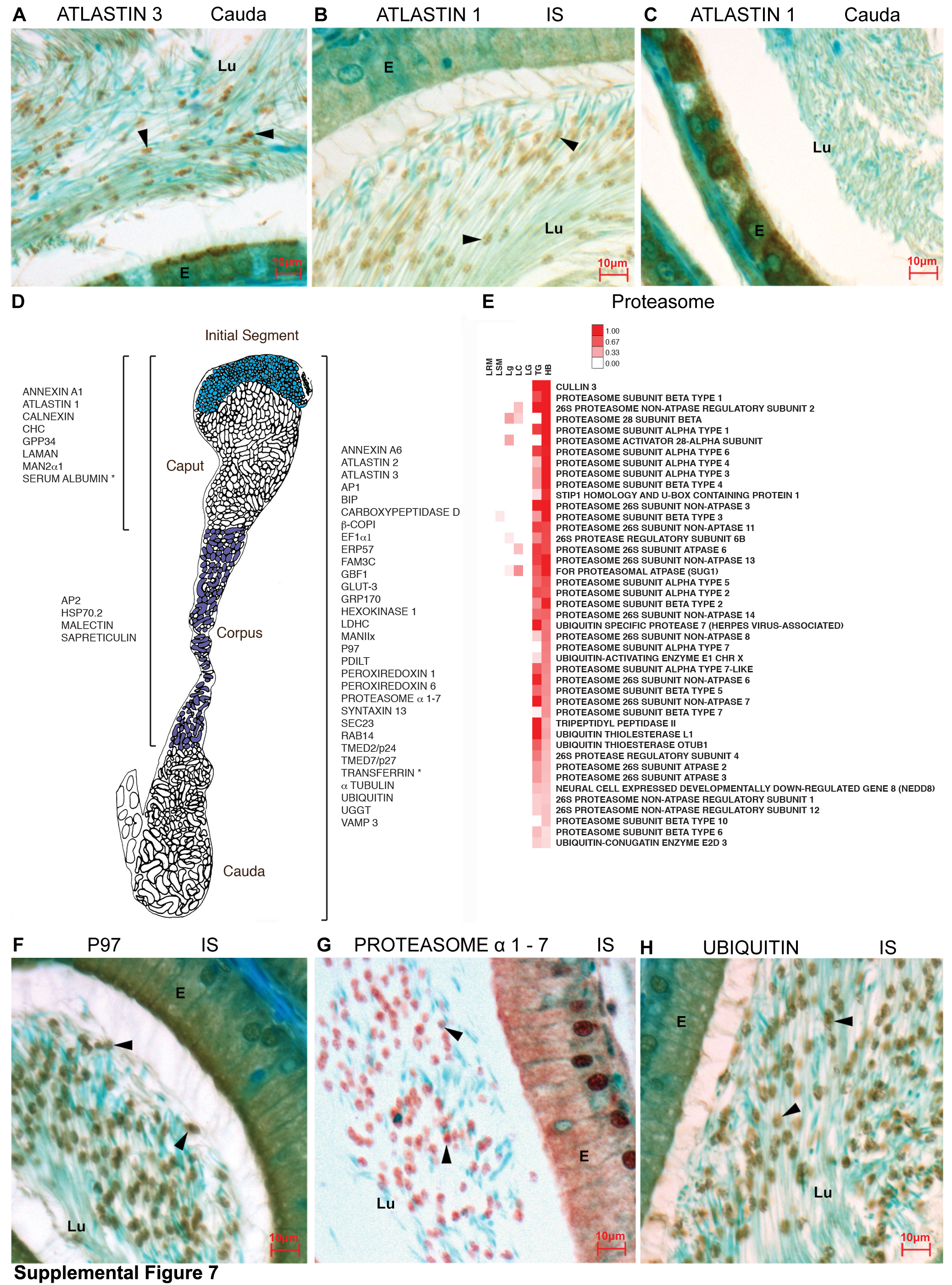

Supplement: HermesSuppFigure-7.tif [file rsob150080supp7.tif]
